# Therapeutic Effect of Photobiomodulation and Single Intratendinous Injection at the Lesion Site of Platelet‐Rich Fibrin in Inducted Tendinopathy

**DOI:** 10.1002/jbio.70271

**Published:** 2026-04-19

**Authors:** Caroline Hammerschmitt Wild, Pâmela Andressa Pauletto, Fransael Franklyn Araújo da Silva, Maria Eduarda Luckner, Gladson Ricardo Flor Bertolini

**Affiliations:** ^1^ Universidade Estadual Do Oeste Do Paraná – UNIOESTE – Cascavel Cascavel Brazil; ^2^ Universidade Federal Do Paraná – UFPR – Palotina Cascavel Brazil

**Keywords:** fibrin, photobiomodulation, physiotherapy, platelets, tendon

## Abstract

Tendinopathy is a multifactorial condition characterized by collagen disorganization, inflammation, and impaired tendon function. Photobiomodulation (PBM) and platelet‐rich fibrin (PRF) have emerged as therapeutic approaches due to their regenerative potential, although their mechanisms during tendon healing remain unclear. This study compared the effects of PBM and PRF, alone or combined, on experimentally induced calcaneal tendinopathy in rats. A total of 75 animals were divided into five groups: control, injury, PRF, PBM, and PRF + PBM. Treatments began 48 h after injury. Functional performance, nociception, and histological features were evaluated up to 21 days. Combined PRF and PBM promoted superior early functional recovery, enhanced collagen synthesis, and increased angiogenesis, despite greater initial inflammation. PBM alone resulted in gradual recovery with better fiber organization, while PRF accelerated function but sustained inflammation. Combined therapy shows promise for tendon repair, supporting stage‐specific treatment strategies.

## Graphical abstract

Combined photobiomodulation and platelet‐rich fibrin accelerate early functional recovery and enhance collagen synthesis and angiogenesis in acute tendon injury, despite increased initial inflammation. PBM alone improves structural organization, while PRF promotes faster recovery, supporting stage‐specific therapeutic strategies for optimized tendon healing.

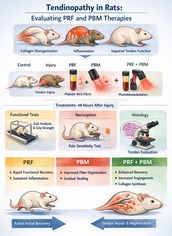

## 1 Introduction

Tendons are fibrous structures responsible for connecting muscles to bones, transmitting force, enabling movement, and contributing to load absorption and proprioception [1]. Its architecture consists mainly of type I collagen organized into microfibrils and fascicles surrounded by endotendons, which house vessels and nerves essential to tissue integrity [2, 3]. In the calcaneal tendon, whose segmented blood supply makes it particularly vulnerable to hypoxia and rupture, the presence of nerve structures such as Golgi tendon organs and C fibers plays an important role in pain perception and inflammatory modulation [4, 5, 6].

Tendinopathy involves microstructural changes, such as collagen fiber disorganization, glycosaminoglycan accumulation, and microvessel proliferation [7]. There is an increase in type III collagen, which is less resistant, predisposing to ruptures [8]. Histologically, there is loss of matrix hierarchy, cellular proliferation, and vascularization, with less inflammatory infiltration in chronic tendinopathies [4].

Clinically, tendinopathy manifests itself through pain, swelling, and dysfunction, and is common in tendons such as the Achilles tendon, patellar tendon, and rotator cuff tendon [9]. Pain can be influenced by excessive nerve growth and neurotransmitters, such as substance P [10, 11], that participate in a peripheral sensitization process characterized by nociceptor hyperactivity, increased sensory fibers in the injured tissue, and release of proinflammatory neuropeptides. These mechanisms amplify pain perception, contributing to the persistence of symptoms even when there is partial reduction of local inflammation. Etiological hypotheses include mechanical overload, poor vascular supply, and myofascial shortening [12]. High intratendinous pressure can activate nociceptors, aggravating pain [13, 14].

Several strategies have been investigated to promote tendon regeneration, including the use of drugs, gene therapies, growth factors, tendon transplants, platelet‐rich plasma applications, and tissue engineering techniques [15]. In clinical physiotherapy practice, physical resources have been studied for the treatment of Achilles tendinopathy, among which photobiomodulation stands out [16, 17].

Photobiomodulation (PBM) works through visible or infrared radiation, stimulating the cytochrome c oxidase complex and increasing ATP production [18, 19]. Its effects include neovascularization, cell proliferation, collagen production, and anti‐inflammatory action, inhibiting mediators such as TNF‐α, IL‐1β, and PGE2 [20]. Studies show benefits in tendon regeneration, although clinical results are still inconclusive [4].

Platelet‐rich fibrin (PRF) is an autologous concentrate containing platelets, leukocytes, and proteins, promoting prolonged release of growth factors such as TGF‐β, PDGF, and VEGF [21, 22]. Unlike PRP, it does not require external activators and forms a manipulable gel [23]. Its variations include L‐PRF, A‐PRF, i‐PRF, C‐PRF, and P‐PRF, with different properties and clinical applications [24, 25].

PRF stimulates tissue regeneration and cell differentiation, with evidence of clinical and histological improvement, although it may cause disorganized healing in some cases [26, 27, 28]. The association between platelet concentrates and PBM represents a promising approach, combining biochemical stimulation and mitochondrial reorganization, although it has been little explored in the literature [29, 30, 31, 32]. However, there is still a significant gap in understanding how these therapies interact in tendon repair, which justifies the need to investigate their combined effects.

Given the complexity of tendon regeneration and the limitations of conventional therapies, this study aims to evaluate the effects of combining PRF and PBM on calcaneal tendon healing, seeking to understand the mechanisms involved and contribute to the development of more effective and personalized therapeutic strategies.

## 2 Methods

### 2.1 Experimental Design

This experimental, quantitative, and descriptive study was approved by the Animal Use Ethics Committee of the State University of Western Paraná with opinion No. 12–23. The experiment was conducted at the LABEF and LABEM laboratories of the same institution. Because animals were euthanized at different time points, evaluations represent independent cross‐sectional samples rather than repeated measures.

### 2.2 Animals

A total of 75 8‐week‐old Wistar rats from the Central Animal Facility of the State University of Western Paraná were used. The animals were kept in a climate‐controlled environment (22°C ± 2°C) with a 12‐h light/dark cycle and received pellet feed and water ad libitum. After acclimatization and adaptation to the functional test, they were randomly distributed into five experimental groups (*n* = 15 per group), with an equal proportion of males and females: Control Group (CG), Injury Group (IG), PRF Group (PRF), PBM Group (PBM), and PRF + PBM Group (PRF + PBM). At D7, D14, and D21, five animals per group were allocated randomly by lottery (https://www.graphpad.com/quickcalcs/randomize1/) to each time point, forming independent subgroups.

### 2.3 Induction of Tendinopathy

Tendinopathy was induced by mechanical clamping of the right Achilles tendon with Halstead forceps on the first tooth of the rack for 2 min, adapted from the protocol by Carvalho et al. [33]. The animals were previously anesthetized with ketamine hydrochloride (75 mg/kg) and xylazine hydrochloride (15 mg/kg), administered intraperitoneally. The procedure was performed under antiseptic conditions with chlorhexidine, with the day of induction considered as D0. This model represents an acute crush‐induced injury rather than overload‐induced tendinopathy.

### 2.4 Preparation of Platelet‐Rich Fibrin (PRF)

PRF was obtained according to the technique described by Choukroun et al. [34]. A total of 1.5 mL of blood was collected by cardiac puncture [35] from each animal in the PRF and PRF + PBM groups using a 22G hypodermic needle. The samples were centrifuged at 3000 rpm (≈400 G) for 10 min, resulting in three layers: platelet‐poor plasma (PPP), PRF, and red blood cells. The PRF was removed with a sterile 22G needle and applied to the calcaneal tendon 48 h after injury (D2). PRF was administered as a single intratendinous injection (1 mL, 22G needle) at the lesion site with standardized depth and needle positioning.

### 2.5 Photobiomodulation (PBM)

PBM was applied to the PBM and PRF + PBM groups using low‐intensity laser, continuous red light (660 nm), power of 30 mW, irradiance of 107.14 mW/cm^2^, spot area of 0.28 cm^2^, 2.52 J per therapy. Each point received 0.84 J (~28 s), totaling 2.52 J per session. Three points (distal, medial, and proximal) of the calcaneal tendon were irradiated using a non‐contact technique at a distance of 0.5–1 cm. The sessions took place on days D2, D4, and D6.

### 2.6 Evaluations

The evaluations were performed on days D0, D7, D14, and D21. On day D0, all animals underwent functional and nociception analyses. On subsequent days, five animals per group were evaluated for functionality, nociception, and histology. Measurements at later time points were independent and not repeated measures.

#### 2.6.1 Functional Assessment

Tendon function was assessed using the Achilles Functional Index (AFI) [36], using images of the animals walking on a treadmill with a camera attached. The measurements were analyzed with Image Pro‐Plus 6.0 software, using the PLF, TSF, and ITF parameters to calculate the index. AFI calculations used the second stride; evaluations at later time points used independent subgroups.

#### 2.6.2 Nociceptive Assessment

Nociception was assessed using digital Von Frey equipment, applying increasing stimuli to the plantar region of the right paw until reflex withdrawal, recording the nociceptive threshold in grams.

#### 2.6.3 Histological Evaluation

The animals were euthanized with ketamine hydrochloride (180 mg/kg) and xylazine hydrochloride (30 mg/kg) administered intraperitoneally. The tendons were fixed in 10% buffered formalin, dehydrated, embedded in paraffin, and subjected to longitudinal microtomy (7 μm). The slides were stained with hematoxylin–eosin (HE) for general morphological analysis and picrosirius red for evaluation of collagen composition under polarized light.

Histological variables were assessed using semi‐quantitative scores ranging from 0–3. Fiber organization was scored from well‐aligned, parallel fibers (0) to completely disorganized bundles without a defined pattern (3). Inflammation and neovascularization were scored according to the percentage of the field occupied by inflammatory cells or vessels, respectively: < 10% (0), 10%–20% (1), 20%–30% (2), and > 30% (3). Two sections per tendon and five non‐overlapping fields per section were analyzed, and all evaluations were performed by a blinded examiner.

### 2.7 Statistical Analysis

Data were expressed as mean ± standard deviation. Generalized linear models (GLM) were used to analyze functional, nociceptive, and histological parameters, followed by Bonferroni post hoc tests, with a significance level of 5%, with the different time points and subgroups being compared independently, that is, without repeating data from the same animals across the different time points. For the functional and nociceptive analyses, the gamma distribution model was used, whereas for the histological analyses, the linear distribution model was used, always based on the lowest AIC (Akaike Information Criterion) value. All analyses were performed using Jamovi 2.3.28 software.

## 3 Results

**FIGURE 1:**
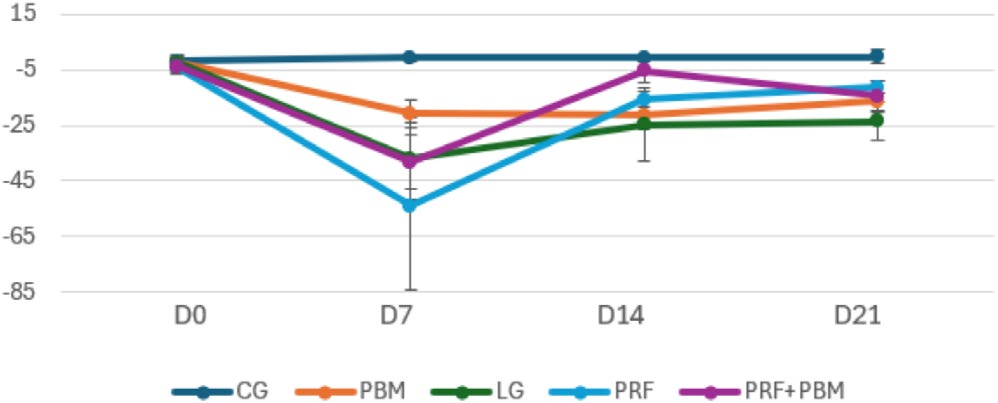
Achilles Functional Index (AFI) of the control (GC), injury (GI), PRF (GPRF), PBM (GPBM), and PRF + PBM (GPRF+PBM) groups at D0, D7, D14, and D21. Data are expressed as mean ± standard deviation. Statistical analysis was performed using generalized linear models followed by Bonferroni post hoc test (*p* < 0.05).

The Achilles Functional Index (AFI) showed that the groups treated with PRF (PRF and PRF + PBM) had earlier functional recovery, with values close to the control group (CG) already at 14 days (Figure 1). The PBM group also showed significant improvement, but later. The injury group (LG) remained functionally impaired until the end of the experiment.

**FIGURE 2:**
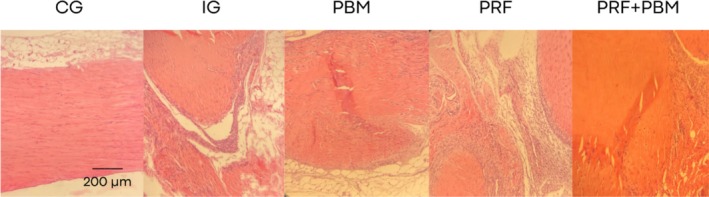
Representative histological sections of the calcaneal tendon of GC, GI, GPBM, GPRF, and GPRF+PBM groups at D7 stained with hematoxylin and eosin (H&E). Images illustrate general tissue morphology, including collagen fiber organization, cellularity, and inflammatory infiltrate. Scale bar = 200 μm.

**FIGURE 3:**
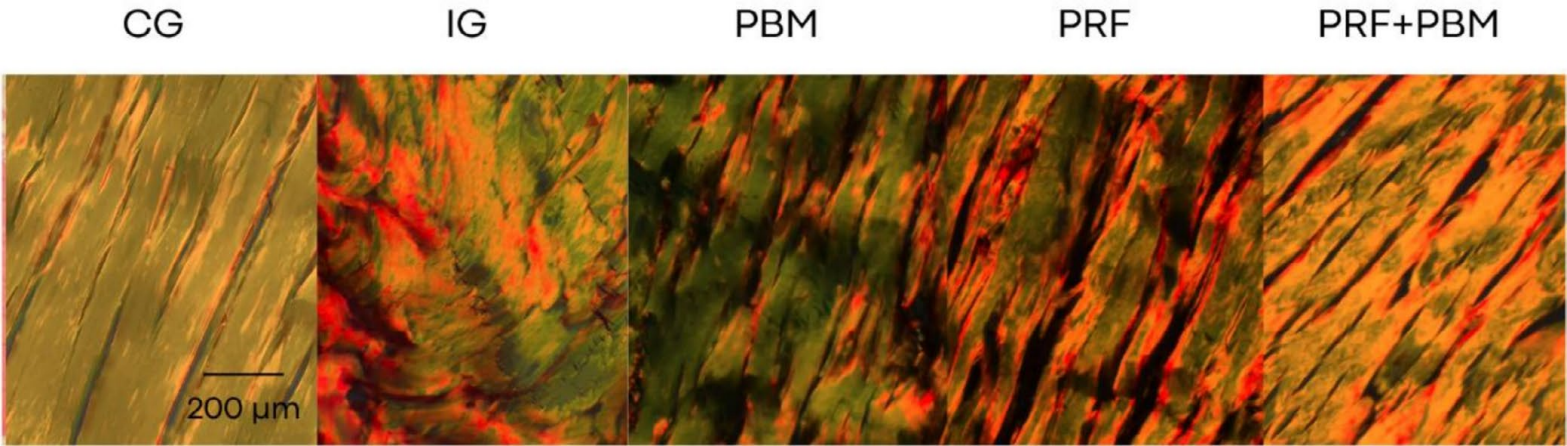
Representative histological sections of the calcaneal tendon of GC, GI, GPBM, GPRF, and GPRF+PBM groups at D7 stained with picrosirius red under polarized light. Images illustrate collagen fiber organization and distribution of type I (red/orange) and type III (green) collagen. Scale bar = 200 μm.

**FIGURE 4:**
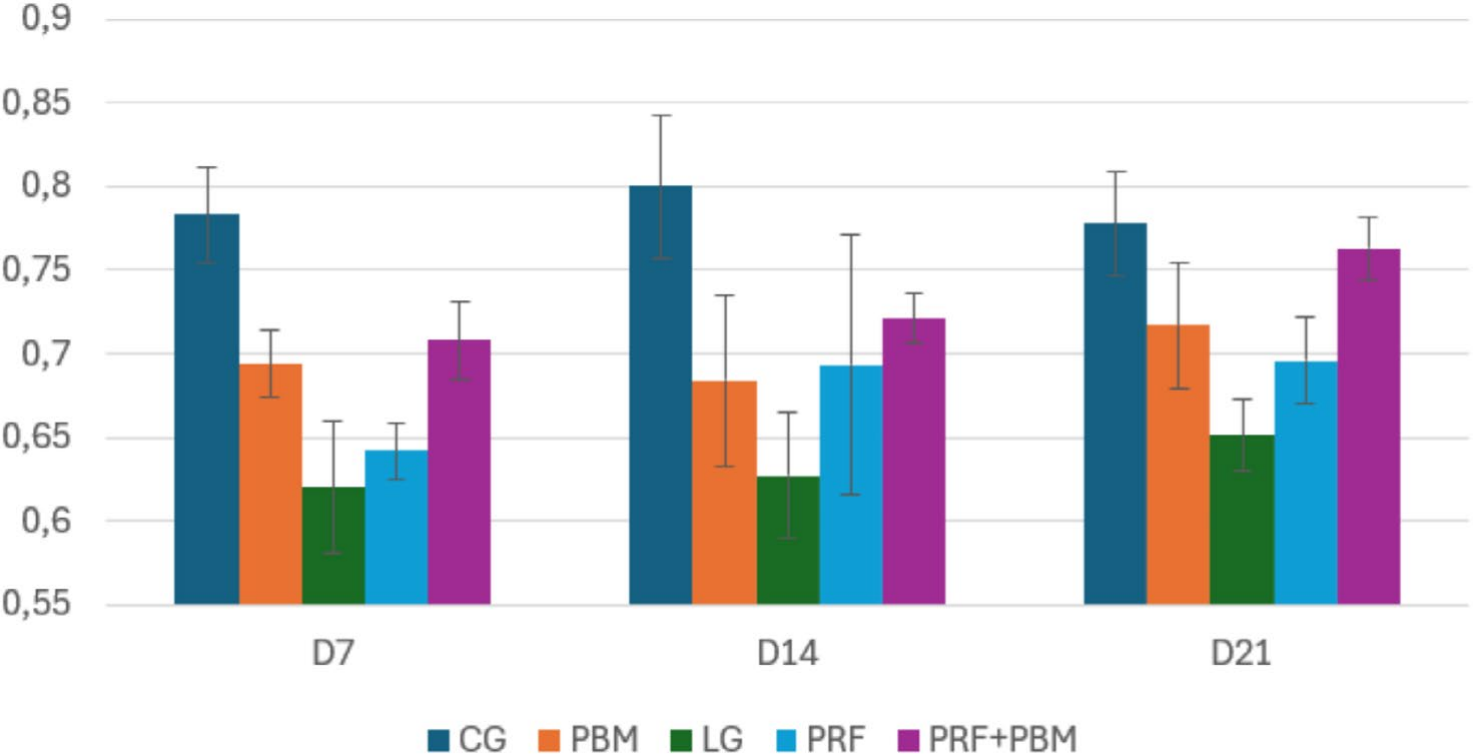
Proportion of type I and type III collagen in the calcaneal tendon of GC, GI, GPRF, GPBM, and GPRF+PBM groups at D7, D14, and D21. Data are expressed as mean ± standard deviation (*n* = 5 per group/time point), obtained from picrosirius red staining under polarized light.

**FIGURE 5:**
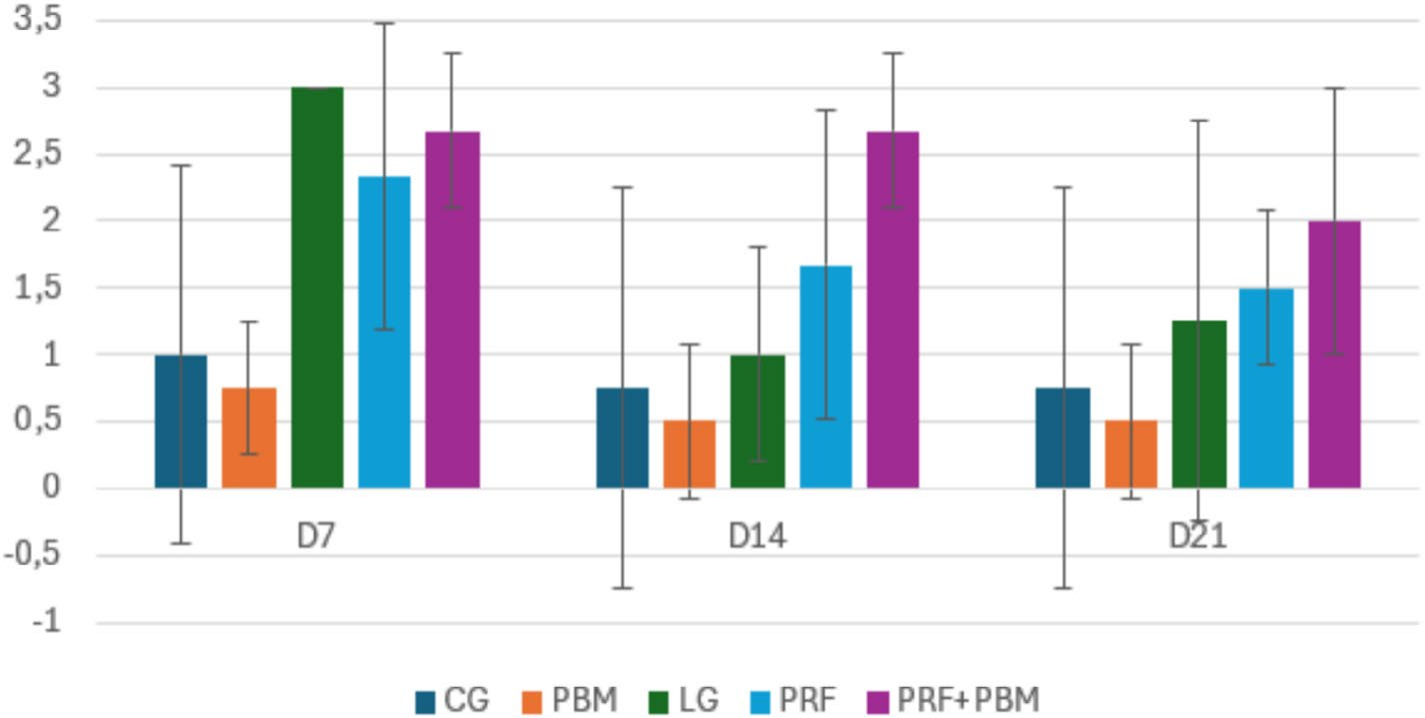
Tendon fiber structure score (0–3) in GC, GI, GPRF, GPBM, and GPRF+PBM groups at D7, D14, and D21. Data are expressed as mean ± standard deviation (*n* = 5 per group/time point). Higher scores indicate greater structural disorganization.

**FIGURE 6:**
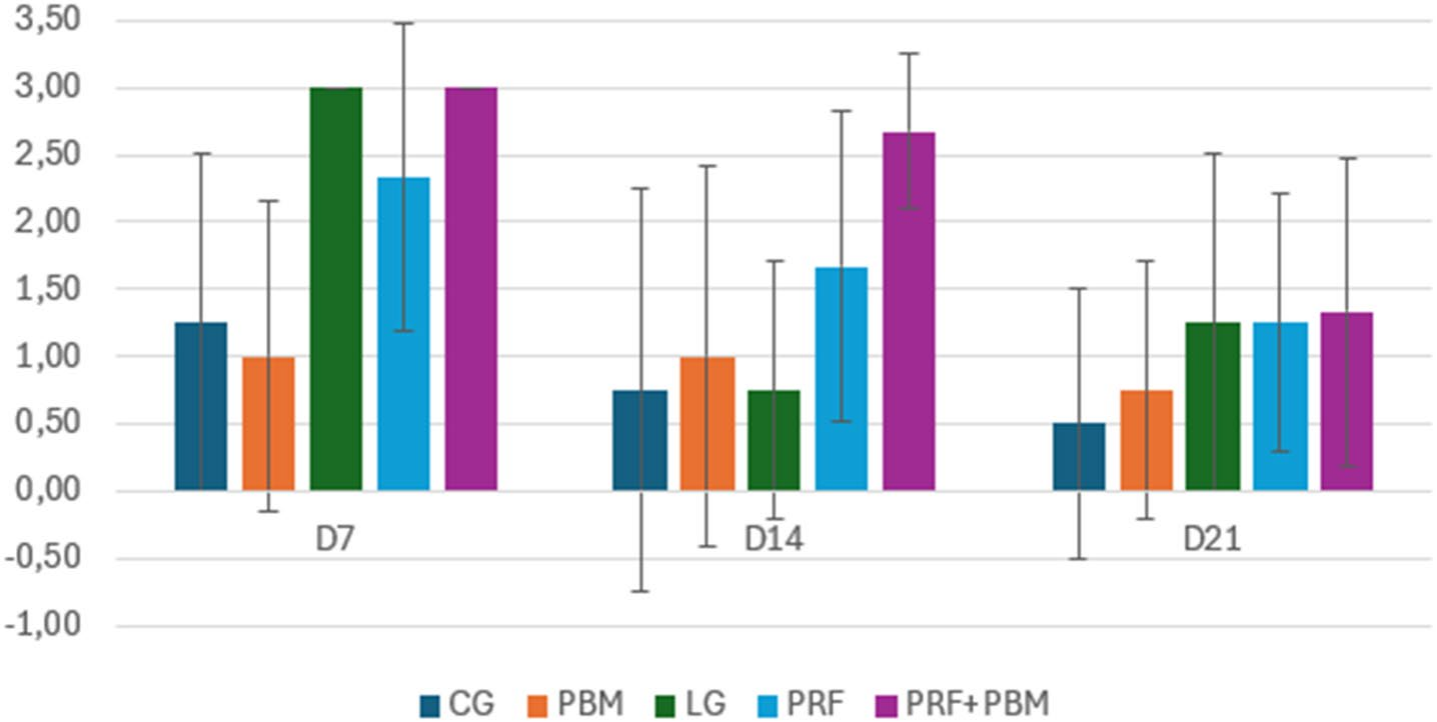
Tendon fiber arrangement score (0–3) in GC, GI, GPRF, GPBM, and GPRF+PBM groups at D7, D14, and D21. Data are expressed as mean ± standard deviation (*n* = 5 per group/time point). Higher scores indicate greater disorganization of collagen fibers.

Representative histological sections stained with hematoxylin–eosin and picrosirius red at D7 are shown in Figures 2 and 3. Histological analysis revealed a progressive increase in the proportion of type I and type III collagen over time, particularly in the PRF + PBM group at D21 (Figure 4). The structure and arrangement of tendon fibers showed greater disorganization in the PRF‐treated groups, especially when associated with PBM, while the PBM‐only group exhibited better fiber organization (Figures 5 and 6).

**FIGURE 7:**
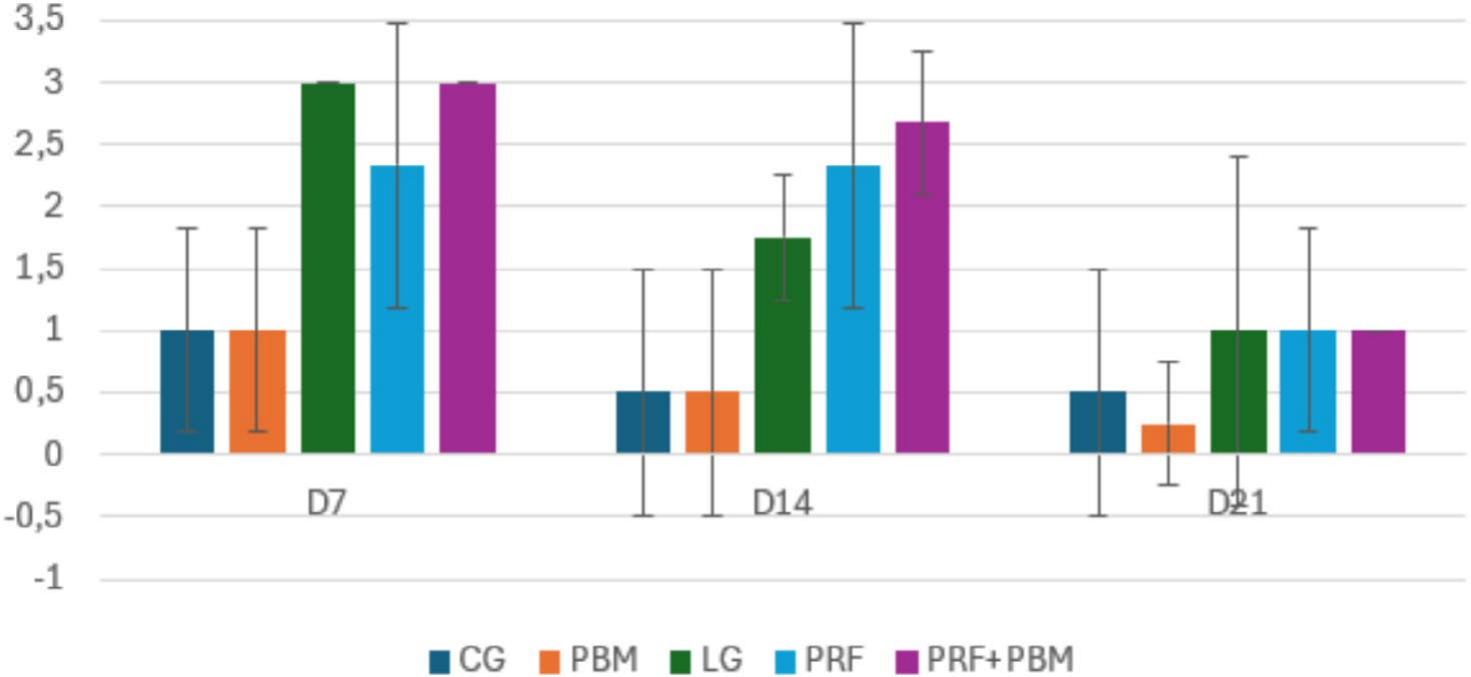
Tenocyte nuclear rounding score (0–3) in GC, GI, GPRF, GPBM, and GPRF+PBM groups at D7, D14, and D21. Data are expressed as mean ± standard deviation (*n* = 5 per group/time point). Higher scores indicate increased cellular activation.

**FIGURE 8:**
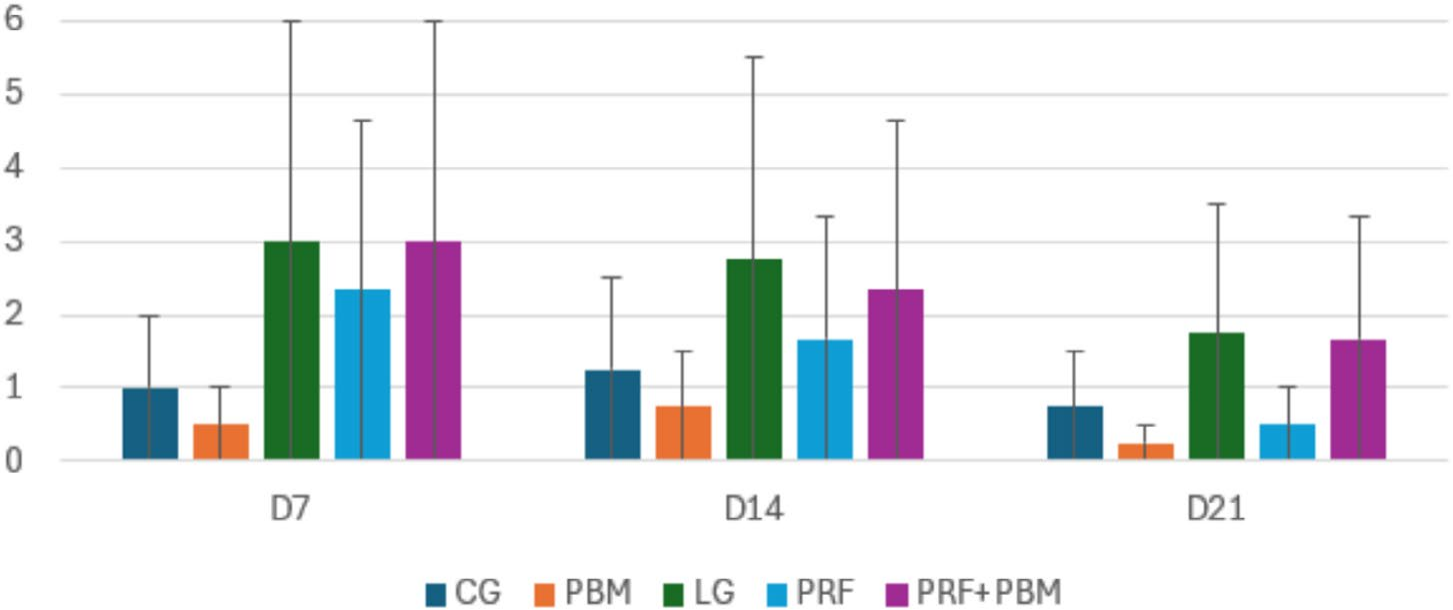
Inflammatory infiltrate score (0–3) in GC, GI, GPRF, GPBM, and GPRF+PBM groups at D7, D14, and D21. Data are expressed as mean ± standard deviation (*n* = 5 per group/time point). Higher scores indicate greater inflammatory cell infiltration.

The nuclear rounding of tenocytes was more evident in the PRF and PRF + PBM groups, indicating greater cell activation and inflammatory response (Figure 7). The area infiltrated by inflammatory cells showed similar behavior among the tissue parameters evaluated. The Injury (LG), PRF, and PRF + PBM groups exhibited the highest inflammatory scores, especially on D7, evidencing the peak of the acute inflammatory response after injury induction. From D14 onwards, there was a gradual reduction in inflammatory infiltrate in all groups, although the groups treated with PRF still had higher values than the others, indicating a more prolonged cellular response (Figure 8).

**FIGURE 9:**
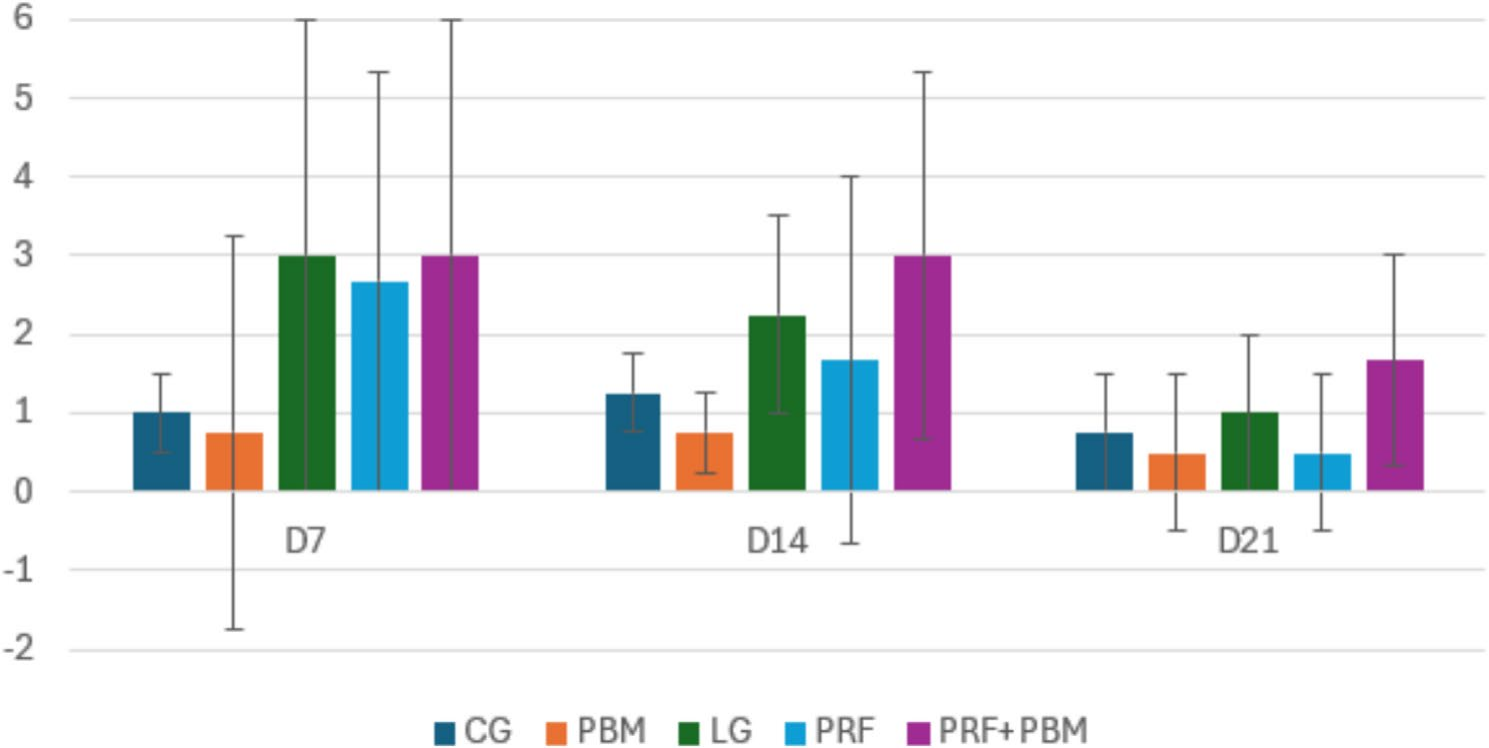
Neovascularization score (0–3) in GC, GI, GPRF, GPBM, and GPRF+PBM groups at D7, D14, and D21. Data are expressed as mean ± standard deviation (*n* = 5 per group/time point). Higher scores indicate increased formation of new blood vessels.

**FIGURE 10:**
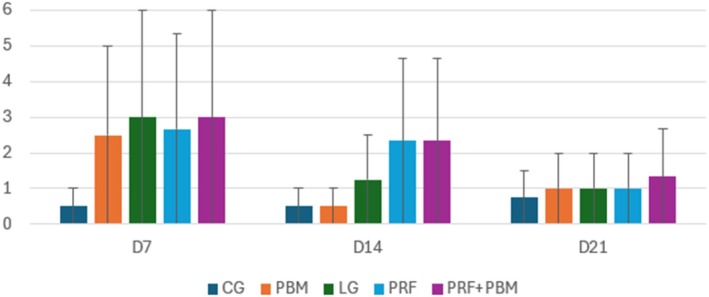
Cell density score (0–3) in GC, GI, GPRF, GPBM, and GPRF + PBM groups at D7, D14, and D21. Data are expressed as mean ± standard deviation (*n* = 5 per group/time point). Higher scores indicate increased cellularity.

Neoangiogenesis was more intense in the groups treated with PRF, peaking on D7, especially in the combined group (PRF + PBM) (Figure 9). Fibroblast density followed a similar pattern, with higher concentrations in the treated groups on D7 (Figure 10). The nociceptive assessment showed no statistical differences between groups or time points.

## 4 Discussion

The present study evaluated the individual and combined effects of PRF and PBM in an acute crush‐induced Achilles tendon injury model, clarifying that the experimental condition reflects a traumatic lesion rather than a chronic overload‐related tendinopathy. This distinction is essential, as crush injuries produce abrupt disruption of collagen architecture, intense early inflammation, and rapid neovascularization, which differ substantially from the progressive degenerative process typical of chronic tendinopathy. Consequently, the findings reported here should be interpreted within the context of acute tendon trauma and not directly extrapolated to chronic clinical tendinopathies.

The application of PRF promoted faster functional recovery compared to PBM, a result that can be attributed to the immediate and intense release of growth factors such as PDGF, TGF‐β, and VEGF [37, 38]. These factors are known to stimulate angiogenesis, cell proliferation, and collagen synthesis, accelerating the formation of initial, albeit disorganized, scar tissue [39]. This rapid response was evidenced by the Achilles Functional Index (AFI), which demonstrated early improvement in tendon function in animals treated with PRF.

Despite accelerated functional recovery, histological analysis revealed that tissue repaired with PRF showed greater inflammation and less organized collagen fibers. This finding suggests that functional gain did not necessarily reflect the structural quality of the repair, corroborating studies that point to immature structural repair in the early stages of PRF‐induced healing [37].

In contrast, PBM demonstrated a more modulating and gradual profile, with less inflammation and better organized collagen fibers over time, indicating a more controlled and efficient repair. The literature reinforces that PBM acts in the modulation of oxidative stress and the reorganization of fibers [40, 41], promoting the deposition of type I collagen and enhancing the strength of repaired tissue [40]. This therapeutic approach, although slower in functional terms, results in tissue with higher structural quality, which is essential for the mechanical strength of the tendon.

Collagen production over time confirmed the natural progression of healing, with a significant increase between days 7 and 21. The control group (CG) had the highest levels of collagen fibers, while the lesion group (LG) had the lowest, evidencing the compromise of the extracellular matrix. PRF promoted a significant increase in collagen synthesis, but with a predominance of disorganized fibers, possibly due to the intense initial inflammatory response [39]. The PBM group, on the other hand, presented lower total levels, but with more aligned and parallel fibers, suggesting better structural and functional integration [42].

However, nociceptive assessment revealed no statistical differences between groups and time points, indicating that the animals' mechanical pain threshold was not significantly modulated by the treatments. This result is consistent with studies showing that pain in tendinopathies does not correlate directly with tissue regeneration [43]. Nociception involves complex mechanisms, such as peripheral and central sensitization, mediated by inflammatory cytokines and neurogenic factors, which may not have been sufficiently altered by the therapeutic protocols applied [44, 45]. Furthermore, the acute injury model used in this study may not have been sufficient to induce persistent nociceptive alterations detectable by this method.

The evaluation of neoangiogenesis revealed that the group treated with PRF associated with PBM showed greater formation of new blood vessels in the initial phase. This synergistic effect can be explained by the combination of PRF growth factors with mitochondrial stimulation induced by PBM [37], which increases the production of ATP and nitric oxide (NO), both related to angiogenic stimulation. However, at later stages, the vascularization of the combined group approached the pattern of the lesion group, which can be attributed to the tissue remodeling phase, in which angiogenesis tends to regress [46].

Finally, analysis of the nuclear morphology of tenocytes reinforced the findings related to the inflammatory response. In normal tendons, the nuclei are elongated, following the axis of the collagen fibers. After injury, they become rounded, indicating cell activation and inflammation [42]. This pattern was observed in the PRF and PRF + PBM groups, suggesting greater initial inflammatory aggressiveness, but also greater stimulation of cell proliferation and collagen synthesis. In contrast, the PBM group maintained more elongated nuclei, evidencing a modulating effect on inflammation and favoring more controlled repair.

Given the results obtained, it can be concluded that PRF promotes faster functional recovery, favoring early restoration of tendon mobility, while photobiomodulation stands out for the structural quality of the repair, with less inflammation and better organization of collagen fibers. The combination of PRF and PBM demonstrated a synergistic effect on initial angiogenesis, although this benefit was not maintained in later stages. Thus, although PRF has advantages in the acute phase of healing, PBM appears to be more effective in promoting lasting and biomechanically more efficient tendon repair. These findings suggest that the therapeutic choice should consider the stage of the injury and the desired clinical objectives, and may even favor combined approaches in personalized protocols.

## Funding

This work was supported by Coordenação de Aperfeiçoamento de Pessoal de Nível Superior.

## Ethics Statement

The research project was approved by the Animal Use Committee of the State University of Western Paraná (UNIOESTE), under number 12–23.

## Consent

The authors have nothing to report.

## Conflicts of Interest

The authors declare no conflicts of interest.

## Data Availability

The data that support the findings of this study are available on request from the corresponding author. The data are not publicly available due to privacy or ethical restrictions.
